# What will it take to increase breastfeeding?

**DOI:** 10.1111/mcn.13371

**Published:** 2022-05-09

**Authors:** Sonia Hernández‐Cordero, Rafael Pérez‐Escamilla

**Affiliations:** ^1^ Research Center for Equitable Development EQUIDE Universidad Iberoamericana Mexico City Mexico; ^2^ Department of Social and Behavioral Sciences Yale School of Public Health New Haven Connecticut USA

**Keywords:** breast milk substitutes, breastfeeding, breastfeeding confidence, breastfeeding support, infancy and childhood, maternal nutrition

## Abstract

The introduction for the Supplement in Maternal & Child Nutrition: What will it take to increase breastfeeding? describes the contribution of each of the articles included in this Supplement to the current evidence about the major structural challenges in place to overcome to improve breastfeeding practices, as well as the evidence‐based policies and interventions that can be effective at advancing breastfeeding on a large scale to promote, protect and support breastfeeding.

## Key messages

- Breastfeeding saves lives, improves the short‐ and long‐term health and cognitive development of infants, and the health of their mothers across the globe.
- In most countries, the vast majority of women are choosing to breastfeed but very often cannot do it for as long as they wish because of major structural barriers across layers of the social‐ecological model.
- Caregivers need to learn the skills to manage normal baby behaviours such as crying and fussiness without placing breastfeeding at risk.
- Implementation of well‐coordinated evidence‐based programmes at the facility and community levels through a highly qualified and motivated workforce, as well as with adequate resources, is needed to improve breastfeeding.
- Improving maternity benefits for women working in the formal and informal sectors and enforcing the World Health Organization Code of Marketing of Breastmilk Substitutes are key to improving breastfeeding.
- Implementation science research is needed to translate into large‐scale policy and practice the vast knowledge that we have on what is needed to improve breastfeeding globally.

The importance of proper nutrition and stimulation early in life for brain development, cognitive development, and the short‐, medium‐ and long‐term health and well‐being of humans is well recognized (Britto et al., 2017). The first 1000 days of life, comprising pregnancy, and the first 2 years postpartum, have indeed been identified as a critical time to have a positive lifelong impact on human development (Hawkes et al., 2019). Breastfeeding is an essential element of optimal nutrition during the first 2 years of life because it saves lives, improves the short‐ and long‐term health of infants, and enhances their cognitive development across countries, regardless of their level of economic development (Bartick et al., 2017; Horta et al., n.d.; Li et al., 2022; Victora et al., 2016). Evidence about the constellation of benefits of breastfeeding for women and children and the biological mechanisms explaining them continues to accumulate (Bode et al., 2020; Parul et al., 2021). With advances in science and technology, more is now known than ever before, about the unique immunological, hormonal, and nutritional properties of breastmilk and how breastmilk composition gets tailored to the unique needs of the infants according to the environments surrounding them (“mother–breastmilk–infant triad”) (Bode et al., 2020). This complex system developed, over millions of years of evolution, protects the health and stimulates the optimal development of the child and as it turns out it also protects maternal health. In mothers, epidemiological studies have shown that breastfeeding reduces the risk of hypertension, cardiovascular disease, type 2 diabetes, breast, and ovarian cancer and may reduce the risk of depression (Bartick et al., 2017; Tschiderer et al., 2022; Victora et al., 2016). Hence, optimal breastfeeding practices in the first 2 years is a fundamental component of a healthy diet since birth (i.e., the first food systems (Baker et al., 2021), and is considered to be a triple‐duty action as it reduces the risk of undernutrition, obesity, and dietary related noncommunicable diseases and fosters child development (Pérez‐Escamilla & Segura‐Pérez, 2018). Beyond the individual and family benefits, breastfeeding is also critical for national development and planetary health (Pérez‐Escamilla, 2017).

The World Health Organization (WHO) recommends early breastfeeding initiation (within the first 60 min postpartum), exclusive breastfeeding (EBF) for 6 months, and continuation of breastfeeding until at least 2 years of age once complementary foods are introduced (UNICEF, 2019). Despite all the benefits of breastfeeding and the fact that, in most countries, the vast majority of women are choosing to breastfeed their infants, a significant percentage of mother‐baby dyads do not receive the full benefits of breastfeeding because of major structural societal factors preventing them to breastfeed their babies for as long as they would like (Pérez‐Escamilla, 2020). Even though UNICEF's, 2019 State of the World's Children (UNICEF, 2019) reported global gains in EBF across regions, breastfeeding practices are suboptimal around the world. The vast majority of countries are still far from reaching the WHO's recently updated EBF target for all countries to reach at least a prevalence of EBF of 70% among infants under 6 months old by 2030 (WHO & UNICEF, 2019). Recently, Neves et al. reported that 48.6% of children under 6 months of age in low‐ and middle‐income countries were exclusively breastfed and 81.1% aged 12 months were still being breastfed with strong variations across countries (Neves et al., 2021).

With a breastfeeding‐friendly environment, the vast majority of women are biologically able to successfully breastfeed, and there are very few medical conditions that contraindicate breastfeeding (Pérez‐Escamilla et al., 2019; WHO & UNICEF, 2009).

Globally, women face important barriers, which occur at multiple levels, interfering with their ability to breastfeed for as long as recommended or as long as she and the infant mutually decide. Breastfeeding practices are influenced by socioeconomic, cultural, and individual factors, as well as by the presence or absence of public policies that support, protect and promote breastfeeding (Perez‐Escamilla et al., 2012; Rollins et al., 2016). A common barrier to breastfeeding is poor support from health workers because of poor knowledge and skills for breastfeeding counselling or promoting the use of breastmilk substitutes (BMS) to mothers and endorsement of such products, lack of prioritization of breastfeeding in clinical settings, and competing workloads, among others (Lhotska et al., 2020; WHO, 2020). Another major barrier is poor maternity protection legislation for women working in both the formal and informal sectors, leading to short and unpaid maternity leave, and workplaces without adequate facilities and policies to support breastfeeding. An additional strong barrier is the pervasive aggressive marketing of BMS (Pérez‐Escamilla, 2020; Pérez‐Escamilla et al., 2012). At the individual level, mother and infant psycho‐social attributes and the relationship between mothers and their infants have a strong influence on infant feeding decisions (Rollins et al., 2016).

Breastfeeding is a shared societal responsibility, where we all must contribute to creating an environment that protects, promotes, and supports breastfeeding. Evidence shows that breastfeeding practices can be improved through interventions delivered in different settings, such as health systems, communities, and homes. Interventions delivered through other settings, such as the workplace, have the potential to increase breastfeeding rates, however, more studies are needed (Pérez‐Escamilla et al., 2012; Rollins et al., 2016).

The aim of this supplement is to bring together the most updated evidence on challenges to improve breastfeeding practices worldwide and to identify promising policies and programmes to protect, promote and support breastfeeding on a large scale. New evidence on the influence of baby behaviours and caregiver's infant feeding decisions during the first 6 months of life, is described by Vilar‐Compte and colleagues in a systematic review (Vilar‐Compte,&nbsp;Pérez‐Escamilla, et al., 2022). The systematic review which included studies with different designs (descriptive, cross‐sectional, prospective, and quasi‐experimental), provided consistent evidence that baby behaviours such as infant crying and fussiness are critical in shaping caregiver's decisions on infant feeding practices. Findings call for urgently addressing the need for well‐trained health providers and counseling programmes to provide guidance to parents and caregivers on common baby behaviours and how to properly cope with them while protecting breastfeeding. Pérez‐Escamilla and colleagues (Pérez‐Escamilla et al., 2022), assessed whether introduction of prelacteals (either milk‐based or water‐based fluids introduced during the first 3 days of life) and BMS nowadays also referred to as commercial milk formulas (WHO & UNICEF, 2022) between 4 days and 4 weeks postpartum undermine breastfeeding success. The authors carried out a systematic review and meta‐analysis, including only prospective studies, allowing them to determine the temporal directionality of the associations. Findings showed a strong relationship between prelacteal feeds and negative breastfeeding outcomes, such as shorter EBF duration and cessation of any breastfeeding among infants under 6 months old. The early introduction of BMS was indeed associated with a lower likelihood of any breastfeeding and of EBF in the first 2 months of life and at 3 and 4 months postpartum, respectively. The authors call for the design of evidence‐based interventions to support breastfeeding at the critical early lactation stages involving the production of colostrum, the onset of lactation, and the successful establishment of breastmilk production required for EBF during the first 6 months of life and the continuation of breastfeeding thereafter. The authors proposed multicomponent interventions focusing on health professionals, pregnant women, mothers of young children, fathers, and their families.

It is well known that self‐reported insufficient milk (SRIM) is the main reason women give all over the world for introducing BMS and oftentimes also for stopping breastfeeding altogether. Segura‐Pérez et al. in their systematic review clearly describe the socioeconomic, demographic, cultural, behavioural, and biomedical factors increasing the risk of SRIM and delayed onset of lactation (DOL) (Segura‐Pérez et al., 2022). This review is quite relevant as the extensive literature on the subject has not been summarized and interpreted in this detail before. Segura‐Pérez and colleagues identified multiple modifiable risk factors for SRIM and DOL including maternal early introduction of BMS, overweight or obesity, caesarean section delivery, and poor maternal physical and mental health risk, as well as mother's interpretation of baby fussiness or crying (Segura‐Pérez et al., 2022;). At the health care systems level maternity practices aligned with the Baby‐Friendly Hospital Initiative (BFHI), such as timely breastfeeding initiation and avoiding in‐hospital BMS supplementation and breastfeeding counseling support, were identified as protective factors for both DOL and SRIM. The authors developed conceptual frameworks for SRIM and DOL based on the systematic review findings, to help guide the development of multicomponent and multilevel interventions to prevent SRIM and DOL. Among other things, the frameworks call for strengthening the implementation of the BFHI and the urgent need for interventions aimed at building breastfeeding self‐confidence in mothers, particularly among primiparous, women.

The second set of evidence presented on this supplement, clearly demonstrates that the widespread, indiscriminate, aggressive, and irresponsible promotion of BMS to which women are exposed across the world, remains one of the biggest challenges to promote, protect, and support breastfeeding. Becker et al., conducted an innovative systematic scoping review that clearly documented widespread violations from the BMS industry to the International Code of Marketing of Breastmilk Substitutes (the Code), while marketing their products directly and indirectly to caregivers across settings and environments surrounding caregivers and their infants including health care systems, public spaces, point of sales such as pharmacies and supermarkets multiple media channels, and humanitarian emergency programmes (Becker et al., 2022). The authors highlight the new BMS promotion strategies such as emergence of new products and marketing practices through social media to circumvent the Code. Two articles in this supplement (Sheikh et al., 2022; Vilar‐Compte, Hernández Cordero, et al., 2022) describe inappropriate BMS promotion practices in Bangladesh and in Mexico, respectively. Sheikh et al., document, for the first time in Bangladesh, the prevalence of Code violations in small grocery stores, supermarkets or grocery chain stores, baby stores, and also through traditional and social media. Vilar‐Compte and colleagues address in their paper how the aggressive and unethical promotion of commercial milk formulas, specifically the follow‐up formulas (FUFs—for children 6–12 months) and growing‐up formulas (GUMs—for children 12–36 months), have become a major threat to advancing breastfeeding in Mexico. This study specifically documented that many Mexican pregnant women and mothers of children younger than 18 months, were very aware of FUFs and GUMs. Furthermore, women who were aware of these commercial milk formula products believed that children from 1 to 3 years need them for helathy growth and development. Both papers highlight that unrestricted promotion of BMS, including FUFs and GUMs, to the public and healthcare professionals needs to be urgently addressed through enforcement of the Code including the implementation of a conflict of an interest‐free monitoring system of Code violations for FUP and GUM products.

The third set of papers on this supplement identified how to improve breastfeeding protection, promotion, and support through evidence‐based interventions and the key enablers needed to successfully scale them up to the national level. Tomori and colleagues updated the evidence of what works to protect, promote, and support breastfeeding on a large scale (Tomori et al., 2022). The authors carried out a review of reviews, highlighting the growing body of literature on effective interventions for addressing breastfeeding barriers across the different layers of the social‐ecological model, including workplace breastfeeding support policies; implementation of the BFHI including skin to skincare; Kangaroo Mother Care; and cup feeding in health settings. The authors also identified the importance of continuity of breastfeeding care and support across health facilities and community and family settings via home visits delivered by community health workers, and support from fathers and grandmothers and other community members. The authors highlight the inadequate attention to interventions addressing policy and structural factors, and workplace settings, and stress the need for rigorous assessment of multicomponent and multilevel interventions, through a breastfeeding equity socio‐ecological lens. Tomori et al. also identified several methodological issues when studying the impact of interventions (Tomori et al., 2022). First, almost all the evidence comes from high‐ and upper‐middle‐income; and the bulk of the interventions evaluated are concentrated in healthcare settings.

The supplement concludes with a comparative analysis of case studies from four countries led by Hernandez‐Cordero and colleagues that identified the different pathways that different countries have taken to successfully scale up breastfeeding programmes and the corresponding enabling factors (Hernández‐Cordero et al., 2022). Through a qualitative thematic analysis, guided by the “Breastfeeding Gear Model” (Pérez‐Escamilla et al., 2012) the authors systematically documented key enabling factors that facilitated the scaling up of interventions, policies or programmes to promote, protect and support breastfeeding, and how hindering factor was overcome. On one hand, findings showed that each of the four countries followed different processes and timing to implement and scale‐up strategies. While in the other hand, in all four countries, advocacy, multisectoral political will, financing, research and evaluation, and coordination were key to fostering an enabling environment for breastfeeding. In all studied countries, there was an urgent need to improve maternity protection and regulation of BMS marketing to protect women, mothers, parents, and relatives from aggressive BMS promotion that often times violates the WHO Code.

In conclusion, this supplement documents that even though there are still major structural challenges in place to overcome to improve breastfeeding practices, there are evidence‐based policies and interventions that can be effective at advancing breastfeeding on a large scale. These include strengthening the BFHI, improving maternity benefits and promoting a breastfeeding‐friendly environment at the workplace, and enforcement of the WHO Code. The latter will require having monitoring systems free from commercial influence, and significant sanctions when there is documentation of violations related to local Code legislation. It will also require the dissemination of information among health professionals and mothers and pregnant women about the Code, the meaning of conflict of interest and why it is so detrimental to the helath and wellbeing of infants and young children, and inappropriate BMS marketing practices.

Consistent with the Breastfeeding Gear Model (Pérez‐Escamilla et al, 2012), this supplement presents strong evidence of the need to improve the access to high‐quality breastfeeding counseling since pregnancy (i.e., anticipatory guidance) to prevent the unnecessary introduction of BMS and reduce the risk of SRIM and DOL. Health providers and health system practices will need to become much more proficient in understanding common baby behaviours, including crying and fussiness, and how to counsel parents on how to manage their concerns without putting breastfeeding at risk. Furthermore, health care professionals will need to be well trained on the principles of conflict of interest and the harm that they can do to the wellbeing of mothers and infants when they allow themselves to become powerful marketing instruments to the BMS industry.

Although this supplement integrates key evidence to understand what it will take to increase breastfeeding, we still have a long way to go to ensure how to effectively translate this knowledge into effective and sustainable large‐scale breastfeeding programmes. More evidence is needed on how to intervene at the household level, specifically how fathers, grandparents, and other family members and friends can support breastfeeding. By the same token, it is important to conduct implementation science‐based studies to better understand how to scale up breastfeeding‐friendly environments at the workplace, as well as how to achieve full compliance with the WHO Code. Finally, there are inequalities in breastfeeding initiation and continuation, with different breastfeeding patterns by income country level (Victora et al., 2016). We still need to understand how to tailor interventions to the needs of the most vulnerable groups to reduce pervasive inequities in breastfeeding practices and corresponding health and development outcomes. Lastly, a critical issue that the ongoing COVID‐19 pandemic has brought to our attention, is the need for countries to be well prepared to protect, support, and promote breastfeeding when public health humanitarian emergencies arise.

## AUTHOR CONTRIBUTIONS

Sonia Hernández‐Cordero and Rafael Pérez‐Escamilla conceptualized and drafted the first version of the article They both critically reviewed the manuscript. Both authors read and approved the submitted manuscript and contributed equally to the article.

## CONFLICTS OF INTEREST

The authors declare no conflicts of interest.

## Data Availability

No data were generated as part of this supplement introduction.
